# Supplementary material for: Accurate sequence-to-affinity models for SH2 domains from multi-round peptide binding assays coupled with free-energy regression
Source: bioRxiv. 2025 Jan 5:2024.12.23.630085. Originally published 2024 Dec 23. Preprint. [Version 2] doi: 10.1101/2024.12.23.630085 (PMC11703206; doi:10.1101/2024.12.23.630085)
Supplement: 1 [file NIHPP2024.12.23.630085V2-supplement-1.pdf]

bioRxiv preprint doi: https://doi.org/10.1101/2024.12.23.630085; this version posted January 5, 2025. The copyright holder for this preprint (which was not certified by peer review) is the author/funder, who has granted bioRxiv a license to display the preprint in perpetuity. It is made available under aCC-BY 4.0 International license.

SUPPLEMENTAL FIGURES

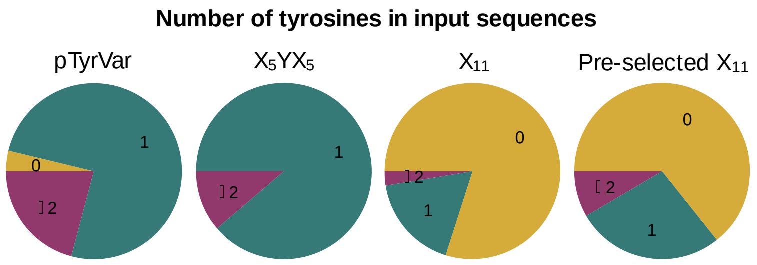

Figure S1: Proportion of sequences containing zero, one, or two or more tyrosine residues in the different input libraries.

bioRxiv preprint doi: https://doi.org/10.1101/2024.12.23.630085; this version posted January 5, 2025. The copyright holder for this preprint (which was not certified by peer review) is the author/funder, who has granted bioRxiv a license to display the preprint in perpetuity. It is made available under aCC-BY 4.0 International license.

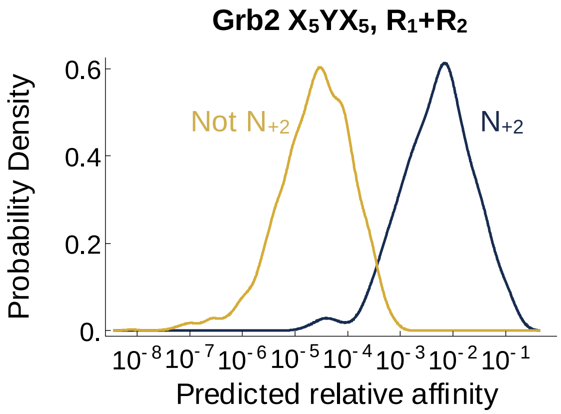

Figure S2: Impact of N_{+2} on predicted Grb2 binding. Plot shows the distribution of binding affinities (shown using a log-scale kernel density estimator) predicted by the Grb2 model in from Fig. 4a. Sequences containing an N_{+2} (blue) are grouped separately from the other sequences (orange).

bioRxiv preprint doi: https://doi.org/10.1101/2024.12.23.630085; this version posted January 5, 2025. The copyright holder for this preprint (which was not certified by peer review) is the author/funder, who has granted bioRxiv a license to display the preprint in perpetuity. It is made available under aCC-BY 4.0 International license.

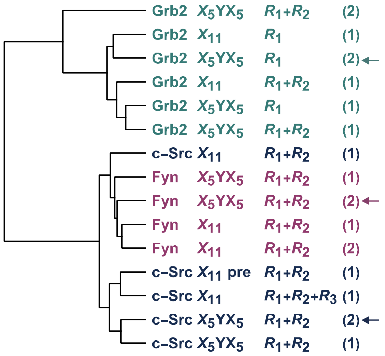

Figure S3: Broader comparison of multi-round models for multiple SH2 domains. The dendrogram shows the clustering of various binding models for the c-Src, Grb2, and Fyn SH2 domains, built using ProBound from data generated using different starting libraries, number of selection rounds. Numbers in parentheses denote replicates. Arrows denote the X_5YX_5 models used for all other analyses in this paper.

bioRxiv preprint doi: https://doi.org/10.1101/2024.12.23.630085; this version posted January 5, 2025. The copyright holder for this preprint (which was not certified by peer review) is the author/funder, who has granted bioRxiv a license to display the preprint in perpetuity. It is made available under aCC-BY 4.0 International license.

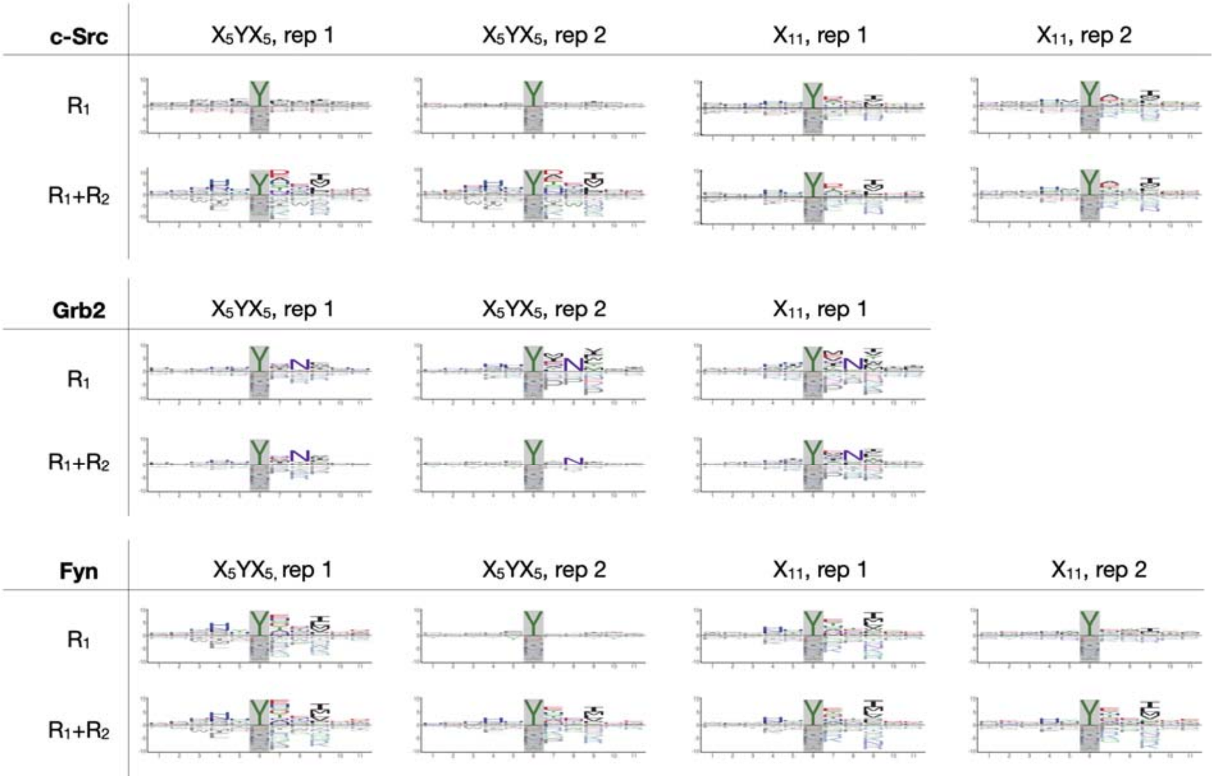

Figure S4: Binding models for the c-Src, Grb2 and Fyn SH2 domains. The models were learned using different combinations of starting libraries (X_5YX_5 or X_{11}), selection round (R_1 or R_1+R_2), and replicates (rep 1 or rep 2).

bioRxiv preprint doi: https://doi.org/10.1101/2024.12.23.630085; this version posted January 5, 2025. The copyright holder for this preprint (which was not certified by peer review) is the author/funder, who has granted bioRxiv a license to display the preprint in perpetuity. It is made available under aCC-BY 4.0 International license.

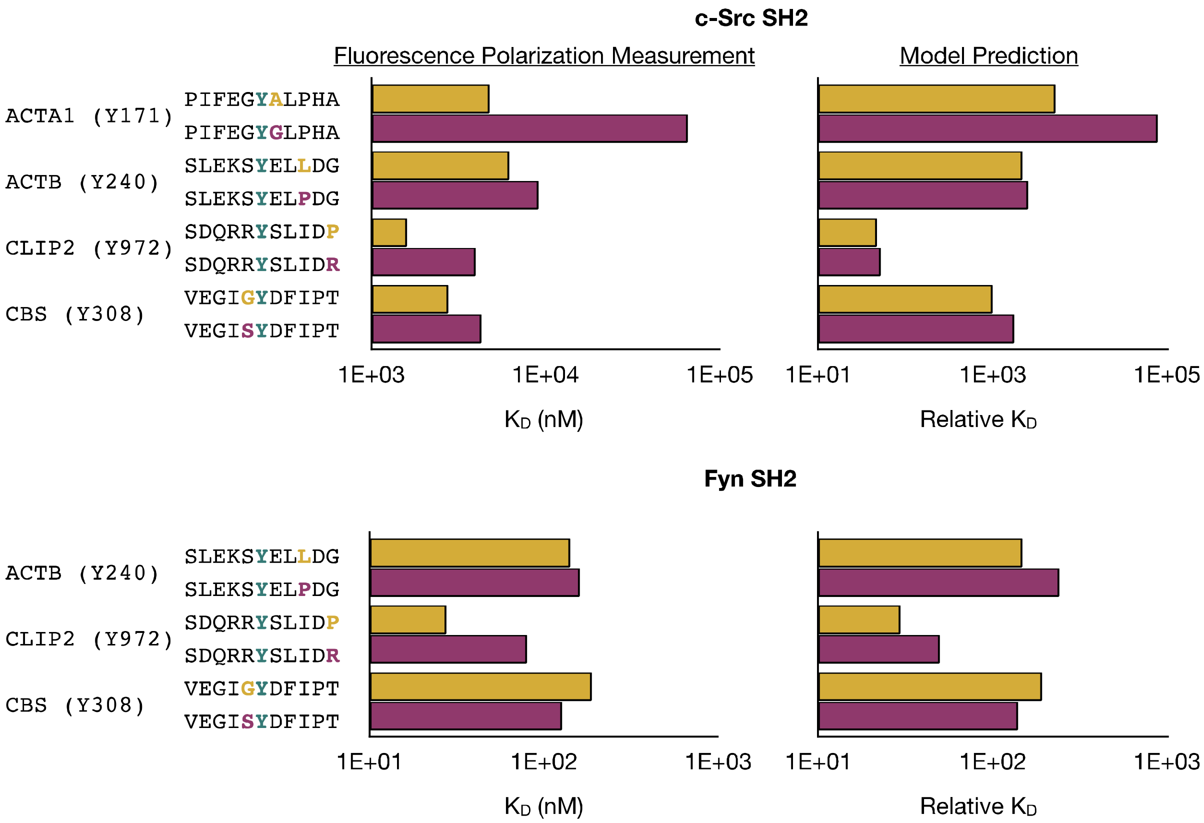

<u>Figure S5</u>: Impact of single-amino-acid substitutions on c-Src and Fyn SH2 binding. The bar charts show the measured K_D value (left) and the predicted relative K_D (right) for pairs of naturally occurring sequence variants (highlighted letters). The predictions were made using the models shown in Fig. 4a.
